# Age differences in socio-emotional feedback processing during learning: an ERP study

**DOI:** 10.3389/fnagi.2026.1756618

**Published:** 2026-04-02

**Authors:** Jana Isabelle Braunwarth, Nicola Kristina Ferdinand

**Affiliations:** Department of Psychology, University of Wuppertal, Wuppertal, Germany

**Keywords:** aging, feedback processing, feedback-related negativity (FRN), P3b, socio-emotionality

## Abstract

In an ever-changing environment, the ability to adapt behavior based on feedback is a crucial skill. Although this process is assumed to decline with age, initial evidence suggests that emotional information processing may help buffer against these age-related impairments. We therefore conducted a probabilistic learning task with emotional faces in two varying emotional intensities (weak vs. strong) to investigate whether healthy younger and older adults would benefit from strong emotional feedback during learning and whether these processes are reflected in feedback-related event related potentials (ERPs). *N* = 26 younger adults (mean age = 22.6, *SD* = 2.67 years) and *n* = 23 older adults (mean age = 71.9, *SD* = 3.60 years) took part in our study. Behavioral results showed that both age groups showed improved learning over time, but older adults particularly benefited from strong emotional feedback. ERPs revealed that the detection of unexpected events, reflected in the peak-to-peak FRN, was generally enhanced in older as compared to younger adults, possibly due to a generally enhanced motivational significance of socio-emotional stimuli. However, we could not find an effect of feedback condition for either group. Moreover, working memory updating, reflected in the P3b, was modulated by emotional intensity in older adults, showing increased updating after strong emotional negative feedback, whereas younger adults did not use strong emotional, but rather negative feedback to update their working memory.

## 1 Introduction

By 2050, one in six people worldwide will be over 65 years old (United Nations, 2023), highlighting the urgency of addressing cognitive aging. In line with this, investigating whether it is possible to reduce age-related learning deficits could help keep older adults engaged and active in society. While age-related declines in executive functions, memory, and reward-system functioning are well-established (Karrer et al., 2017; Salthouse, 2004), emotional processing appears relatively preserved, likely supported by neural mechanisms and a motivational shift toward emotionally meaningful information that together enhance the processing of emotional stimuli (Carstensen, 1991; Carstensen et al., 2006; Leclerc and Kensinger, 2008; Mather, 2012). However, it remains unclear to what extent socio-emotional feedback can lead to learning benefits and reduce age-related declines. This study therefore addresses this gap by examining the influence of socio-emotional feedback of varying intensities on learning in older adults, providing new insights into potential brain processes.

When learning through trial and error, mistakes provide feedback that allows us to adapt our behavior (Holroyd and Coles, 2002). Effective behavioral adjustment requires monitoring performance, detecting unexpected events, and updating working memory (Ullsperger, 2024). The medial prefrontal cortex (mPFC), particularly the dorsal anterior cingulate cortex (dACC), plays a key role in this process by integrating dopaminergic prediction error signals from the midbrain (Alexander and Brown, 2011; Cohen, 2008; Schultz, 2002). These processes can also be indexed in feedback-locked ERPs using an electroencephalogram (EEG). The feedback-related negativity (FRN) is a frontocentral negative deflection around 200–300 ms after feedback, typically generated in the dACC (Miltner et al., 1997; Walsh and Anderson, 2012). It has been interpreted as reflecting the monitoring of outcome salience or unexpectedness, representing an unsigned reward prediction error (Ferdinand et al., 2012; Oliveira et al., 2007; Paul et al., 2025; Pfabigan et al., 2011; Talmi et al., 2013). This interpretation is supported by fMRI findings of increased ACC activation for unexpected compared to expected events (Braver et al., 2001; Ferdinand and Opitz, 2014; Wessel et al., 2012). Other accounts conceptualize the FRN as a signed reward prediction error, specifically signaling outcomes that are worse than expected (Gehring and Willoughby, 2002; Holroyd and Coles, 2002; Sambrook and Goslin, 2014). More recent work additionally highlights the reward positivity (RewP), linked to enhanced processing of positive outcomes (Holroyd et al., 2008; Proudfit, 2015). Overall, the FRN is shaped by several feedback factors, including negative valence, magnitude, expectancy, and timing (Kirsch et al., 2022; Martín, 2012; Opitz et al., 2011; Severo et al., 2020; Yeung et al., 2004). In line with this, the present study adopts the expectancy-violation framework, interpreting the FRN as an index of neural sensitivity to unexpected events. After this initial monitoring stage, a subsequent process facilitates behavioral adaptation by updating working memory. In the ERP, this step is indexed by the P3b, a positive component peaking around 350–500 ms after feedback presentation with a maximum at parietocentral sites (Donchin and Coles, 1988; Martín, 2012; McCarthy and Donchin, 1981; Polich, 2007). The P3b is known to be sensitive to the frequency of events, as well as task complexity and task relevance. Specifically it means that P3b amplitudes are enhanced for rare, unexpected, or behaviorally relevant stimuli, and are further shaped by working memory load, decision uncertainty, and the requirement to update task representations, thereby underscoring the function of the P3b as a marker of contextual updating (Bellebaum and Daum, 2008; Berg et al., 2019; Ferdinand et al., 2012; Mecklinger et al., 1994; Paul et al., 2025; Severo et al., 2020; Ullsperger et al., 2014). This contextual updating has been shown to be enhanced after negative valence in probabilistic learning tasks, suggesting that negative feedback is more relevant to perform these tasks successfully (Ferdinand, 2019; Ferdinand and Hilz, 2020; Frank et al., 2005; Kamp et al., 2024).

Aging is associated with changes in neural systems, including a decline in dopaminergic neurons that support performance monitoring and behavioral adaptation (Baeckman et al., 2000; Bäckman et al., 2006; Karrer et al., 2017; Nieuwenhuis et al., 2005; Schultz, 2002; Ullsperger, 2024). This decline is characterized by reduced receptor density, firing efficiency, and dopamine availability in regions such as the prefrontal cortex and striatum (Bäckman and Farde, 2005; Collier et al., 2011; Lighthall et al., 2018; Wild-Wall et al., 2009). Correspondingly, feedback-learning studies show that older adults are less efficient at detecting unexpected events, as reflected in smaller FRN amplitudes and reduced differentiation between positive and negative feedback compared to younger adults (Bellebaum et al., 2012; Eppinger et al., 2008; Ferdinand, 2019; Ferdinand and Kray, 2013; Hämmerer et al., 2011; Herbert et al., 2011; Nieuwenhuis et al., 2005; Pietschmann et al., 2011; West and Huet, 2020). Regarding working memory updating, P3b amplitudes decrease with age, particularly in non-learning tasks such as oddball or task-switching paradigms (Friedman et al., 2007; Gajewski et al., 2018). Although this reduction is typically interpreted as reflecting age-related decline in updating processes, findings from feedback-learning tasks are mixed: some studies report preserved P3b amplitudes in older adults (West and Huet, 2020), whereas others observe reduced amplitudes (Daffner et al., 2011; Gajewski and Falkenstein, 2014; Polich and Criado, 2006; Wild-Wall et al., 2009). These inconsistencies may be partly explained by task characteristics, such as task complexity and the reliance on strategic updating (e.g., focusing on the most relevant feedback; Ferdinand and Kray, 2013; Gaál and Czigler, 2015).

Moreover, the literature consistently reports age-related topographical changes attributed to reduced working memory capacity and compensatory frontal recruitment, with younger adults showing predominantly parietal activation and older adults exhibiting a more broadly distributed topography including frontal regions (Cabeza et al., 2002; Daffner et al., 2011; Ferdinand, 2019; Ferdinand and Hilz, 2020; Friedman et al., 1997; Lubitz et al., 2017; Pietschmann et al., 2011; Reuter-Lorenz et al., 2000; Schmitt et al., 2014a,b; Tregellas et al., 2006; Wild-Wall et al., 2009). These neurophysiological changes are also reflected in observable behavioral patterns with increased error rates and slower reaction times compared to younger adults (Eppinger et al., 2008; Ferdinand, 2019; Lighthall et al., 2018; Mell et al., 2005; Pietschmann et al., 2011; Wild-Wall et al., 2009). In sum, there is much evidence of changes that appear to impair the efficient processing of feedback, thereby limiting behavioral adaptation in learning from feedback in older adults.

Although cognitive decline is a hallmark of aging, socio-emotional processing seems to remain largely intact. Behavioral findings suggest that older adults perceive and respond to socio-emotional stimuli much like younger adults, indicating that these functions are comparatively resilient to age-related decline (Carstensen et al., 2006; Ferdinand et al., 2018; Ferdinand and Czernochowski, 2018; Kensinger and Gutchess, 2017). Two complementary mechanisms have been proposed to account for this relative preservation. First, neuroimaging studies indicate that key neural systems involved in socio-emotional processing, such as the amygdala and insula, undergo comparatively little structural or functional decline with age (Leclerc and Kensinger, 2008; Mather, 2012). Second, motivational theories of aging, e.g., the socio-emotional selectivity theory (Carstensen, 1991; Carstensen et al., 2006) or the Strength and Vulnerability Integration (SAVI) model (Charles, 2010; Charles and Luong, 2013; Charles and Piazza, 2024), suggest that aging is accompanied by a motivational shift which includes maintaining a positive emotion regulation balance and investing in emotionally meaningful social interactions. To achieve this, a focus on processing socio-emotional stimuli is crucial. Together, these factors suggest that socio-emotional feedback may be particularly effective in supporting learning from feedback in older adults. In line with these considerations, studies with older adults have shown that positive socio-emotional feedback (happy faces) can enhance performance under low task complexity, whereas negative feedback (angry faces) impairs learning when complexity is high (Gorlick et al., 2013). However, when socio-emotional feedback was compared to monetary feedback, no additional benefit of emotional feedback was found (Nashiro et al., 2011).

To our knowledge, the only study examining the electrophysiological correlates of learning from socio-emotional feedback in older adults was performed by Ferdinand and Hilz (2020), who examined whether socio-emotional feedback modulates learning performance and neural feedback processing in younger and older adults. In a probabilistic learning task, participants received either socio-emotional (happy vs. disgusted) or neutral facial feedback (background color indicating correct vs. incorrect responses). Older adults learned better when socio-emotional feedback was provided, likely because negative feedback in this condition triggered stronger working memory updating, as reflected in enhanced P3b amplitudes compared to neutral feedback. No modulation of the FRN by socio-emotional feedback was found. However, this study contained some limitations. First, ERP analyses revealed enhanced FRN peak-to-peak amplitudes after neutral feedback, suggesting that this type of feedback induced large expectancy violations.

This raises the question of whether neutral faces are an appropriate choice for non-emotional stimuli, given their atypicality and potential negative connotation in everyday social interactions. Moreover, in this study, the neutral expressions were presented on blue or yellow backgrounds to signal correct or incorrect feedback (randomized across participants). This design likely added working memory demands in the neutral condition, as participants had to remember the color–feedback associations to be able to learn. Both limitations could have caused worse learning rates and affected the ERPs in the neutral feedback condition. Thus, it is not clear whether the results reflect a processing benefit for socio-emotional feedback or a processing disadvantage in the neutral feedback condition (Ferdinand and Hilz, 2020). In contrast, findings for younger adults do not consistently show improvements after socio-emotional feedback. Hurlemann et al. (2010) investigated facilitation of declarative learning after social relative to non-social stimuli (faces vs. lights). Social feedback (thumps vs. signs) further elicited larger FRN and P3b responses and improved time-estimation accuracy by Pfabigan et al. (2019). In contrast, Ferdinand and Hilz (2020) reported no advantage for younger adults neither on a behavioral nor on a neural level in a probabilistic learning task, possibly due to ceiling effects in the learning task (Ferdinand and Hilz, 2020). Another study by Braunwarth and Ferdinand (2025) therefore manipulated task complexity by increasing working memory load in younger adults and found that socio-emotional feedback facilitated performance in the difficult task after initial task knowledge was acquired. Socio-emotional compared to non-emotional feedback enhanced detection of unexpected events (larger FRN) and working memory updating (larger P3b), irrespective of task complexity, indicating increased sensitivity to socio-emotional feedback (Braunwarth and Ferdinand, 2025). In conclusion, while some advantages of socio-emotional feedback in older adults are evident, the challenge of defining a suitable “neutral” feedback condition remains, leaving the full extent of these benefits unclear and contributing to the mixed findings within age groups so far.

With the present study, we aim to add understanding on how socio-emotional feedback can support learning performance as well as monitoring processes and working memory updating in younger and older adults. Similar to Ferdinand and Hilz (2020), we used a probabilistic learning task, in which stimulus-response associations had to be learned via feedback. To address the limitations of neutral feedback discussed above, we used weak vs. strong emotional feedback. This way, the feedback stimuli became more comparable, and we did not need to use color to signal feedback valence. We assumed that younger adults would learn faster, reflected in a steeper increase in accuracy and decrease in reaction times across the experiment, and would reach overall higher learning rates than older adults. Based on previous findings, we expected ERP effects related to the detection of unexpected events and working memory updating to be primarily driven by negative feedback, thereby replicating previously reported feedback-related ERP modulations. Further, we expected that older adults would show less efficient detection of unexpected events, indicated by smaller peak-to-peak FRN amplitudes compared to younger adults, and reduced working memory updating, indicated by smaller mean P3b amplitudes. However, we assumed that particularly older adults would benefit from strong emotional feedback during learning, as reflected in higher accuracy and faster reaction times in this condition compared to the weak emotional condition. In addition, we expected that detection of expectancy violations (FRN) and working memory updating (P3b) would be enhanced following strong emotional feedback. Finally, we hypothesized that older adults would show stronger frontal activation of the P3b relative to younger adults, reflecting increased compensatory updating demands, particularly for weak compared to strong emotional feedback.

## 2 Methods

### 2.1 Participants

Prior to conducting the experiment, we used G^*^power (Faul et al., 2007) to estimate the minimum sample size using a power of *f* = 0.8, a small to middle-sized effect size of 0.15, which was based on earlier studies (Braunwarth and Ferdinand, 2025; Ferdinand and Hilz, 2020), and an α-level of 0.05. This analysis revealed a minimum of 18 participants for each group for our largest hypothesized interaction. Taking possible drop-outs into consideration, we aimed to recruit a minimum of 25 younger (aged 19–29 years) and 25 older adults (aged 67–80 years). Participants were recruited through social media and the SONA recruiting system at the University of Wuppertal. Older adults were further recruited using flyers distributed in local institutions, sports clubs, and cultural facilities. Participants either received course credit or 10€/h expense allowance. Inclusion criteria were right handedness, no psychiatric or neurological diseases, no pacemakers or cochlear implants, and normal or corrected-to-normal vision. Older adults additionally completed the Mini-Mental State Examination (MMSE) and were included in the study only if they achieved a score >27 (Salis et al., 2023), ensuring the absence of cognitive impairment. Four older and two younger participants were excluded from the final sample due to EEG data with more than 25% artifactual trials, visual impairments, or insufficient understanding of the task requirements. Participants were also tested on the Digit Symbol Substitution Test (DSST; adapted from Wechsler, Jaeger, 2018) and the Multiple choice vocabulary test (MWT-B; adapted from Lehrl, 2005) to asses fluid and crystallized intelligence, respectively. Younger adults outperformed older adults on the DSST [*t* (47) = −6.76; *p* < 0.001, two-tailed], while older adults obtained higher values in the MWT-B [*t* (46) = 8.39; *p* < 0.001, two-tailed]. These findings match the idea of declining fluid and preserved crystallized intelligence with increasing age (Baltes and Lindenberger, 1999).

We followed the Declaration of Helsinki and the study was approved by the ethics committee of the University of Wuppertal. All participants signed informed consent before participation. An overview of the final sample can be found in Table 1.

**Table 1:** Group demographics.

Variables	Older adults	Younger adults
Age	71.6 (3.6)	22.6 (2.7)
Sex *n* (male/female/divers)	9/14/0	13/13/0
First language	German (*n* = 23)	German (*n* = 22)
		Farsi (*n* = 2)
		Arabic (n.s.; *n* = 1)
		Moroccan Arabic (*n* = 1)
MWT score	27.6 (3.1)	20 (3.1)
DSST score	41.5 (8.8)	60.7 (10.9)

MWT, multiple choice vocabulary test; DSST, digit symbol substitution test; n.s., not specified. Values are means with standard deviations in parentheses where applicable.

### 2.2 Task and stimuli

**Figure 1:**
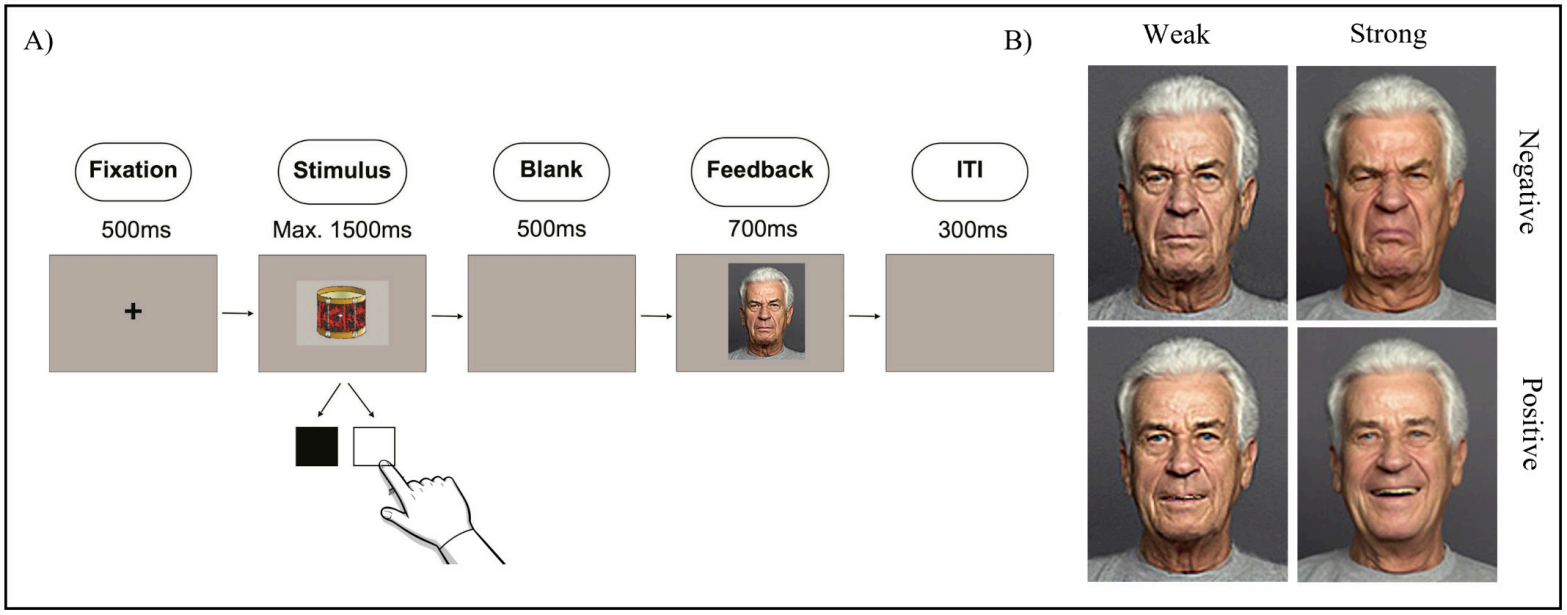
**(A)** Representative trial procedure depicting weak negative emotional feedback. **(B)** Weak (morphed) and strong emotional feedback illustrated for the older man across both valences.

In the present study, participants completed a probabilistic learning task (adapted from Ferdinand and Hilz, 2020), in which they learned a stimulus-response association by pressing a button, followed by feedback (see Figure 1). The stimuli consisted of different object groups (e.g., clothing, fruits, animals) and were taken from a standardized object database (Rossion and Pourtois, 2004). Each object had a size of 281 × 197 or 197 × 281 pixel and was either linked to weak or strong emotional feedback. The feedback stimuli were taken from the FACES database (Ebner et al., 2010) with a size of 112 × 140 pixel. The strong emotional feedback condition consisted of happy faces for positive and disgusted faces for negative feedback, as the ability to recognize those two emotions seems to remain relatively intact as people age (Orgeta and Phillips, 2007; Ruffman et al., 2008). For the weak emotional feedback, the same faces as in the strong emotional condition were used. To reduce emotional intensity, these images were morphed with neutral faces from the same person from the same database using Fantamorph Version 5.0 (FantaMorph, Abrosoft: http://www.fantamorph.com/; Abrosoft, 2016). To identify the most suitable morph level, we conducted a pre-study with *N* = 69 participants (*M* = 24.39 years, *SD* = 6.73), in which 10 different morph levels (90% neutral face morphed with 10% strong emotional face, 80% neutral with 20% strong emotional, …, 10% neutral with 90% strong emotional) were rated with respect to perceived emotionality. The aim was to select images that were clearly distinguished from neutral stimuli, but at the same time contained significantly less emotion than the strong emotional images. Based on these ratings, the weak emotional stimuli were defined as those representing the lowest morph level that was perceived as emotional while also significantly different from the strong emotional condition. As a result, a morph level of 40% emotional face (happy or disgust) and 60% neutral face was identified for both valences and used as weak emotional feedback in the main study. To address potential biases related to age and gender, four distinct persons were used for face stimuli, including a young man, young woman, old man, and old woman, presented in a counterbalanced order across all participants (all four of them were included in the pre-study). All facial stimuli presented were White. Images were presented on a grayscale background and an example image can be found in Figure 1.

In the probabilistic learning task, participants were instructed to act as moving helpers, deciding whether objects had to be loaded in a black or a white truck. Colored response keys on the keyboard (“*c*” for black, “*m*” for white) were used for item allocation and participants received feedback from their boss based on their responses. To avoid one-trial learning, 10% of the received feedback was invalid, resulting in positive feedback after incorrect responses and negative feedback after correct responses. Before the main experiment started, all participants completed eight practice trials, with the option to repeat if necessary. The experiment consisted of 720 trials, which were divided into four learning blocks. Each learning block consisted of 180 trials in which the same six objects of an object category had to be allocated to one of the two keys. Short breaks were provided every 60 trials, with participants determining their duration. After each learning block, we included a short retrieval task asking about the assignment of the objects by showing every object again and asking the participants to press the correct button. At the start of a trial, a fixation cross appeared for 500 ms followed by an object starting with a maximum response window of 1,000 ms in the first trial. To counteract age and individual differences in the speed of the task, the maximum response time was adapted (in the range of 600–1,500 ms) based on participant's timeouts. In case of exceeding the response window, participants received a message on the screen saying “zu langsam” (German for “too slow”). Participants who did not exceed the time range within 20 trials received a response time of 100 ms less for the next 20 trials. If they exceeded the time once in these trials, the time range remained the same for the next 20 trials. In case of exceeding the time window more than once, they were given 100 ms more in the next 20 trials. Afterwards, a blank screen appeared for 500 ms and then the feedback was shown for 700 ms followed by an intertrial interval of 300 ms (see Figure 1).

### 2.3 Procedure

Upon arrival, participants signed informed consent. Older adults underwent the MMSE (Folstein et al., 1975) and both groups right-handedness was assessed using the LQ-Questionnaire (Oldfield, 1971). They then completed the Digit Symbol Substitution Test (Jaeger, 2018) as a measure of fluid intelligence and a demographic questionnaire covering education, exercise, nutrition, and social participation. Additional questionnaires included assessments of emotion regulation (Abler and Kessler, 2011) and the German version of the Interpersonal Reactivity Index (Paulus, 2009). Participants also completed a digital version of the german MWT-B to measure crystallized intelligence (Lehrl, 2005). After EEG preparation, the probabilistic learning task was presented on a 27 inch screen in an electrically shielded sound proof EEG cabin. Afterwards, participants answered follow-up questions on sleep, concentration, and learning strategies before receiving expense allowances and departing.

### 2.4 EEG recording and analysis

While EEG was recorded using BrainVision Recorder 2.0 (BrainVision Recorder, Version 1.23.0003, Brain Products GmbH, Gilching, Germany), the experimental paradigm was presented by E-Prime 3.0 (Psychology Software Tools, Pittsburgh, PA). 58 active silver/silver chloride electrodes were attached according to the international 10–20 system (Jasper, 1958). The ground electrode was placed at position AFz and the left mastoid served as online reference electrode. An electrooculogram (EOG) was recorded for offline eye movement correction. For this purpose, electrodes were placed supra- and infraorbitally to the right eye and near the outer canthi of both eyes. All impedances were kept below 20 kΩ. The EEG and EOG signal were filtered online using a low pass filter (250 Hz) and digitized with a sampling rate of 500 Hz. Offline processing was performed using Matlab R2023 (The Mathworks Inc., Natick, Massachusetts, USA) (The Mathworks Inc., 2022) and the eeglab toolbox v.2021.1 (Delorme and Makeig, 2004). A high pass filter of 0.01 Hz and a low pass filter of 30 Hz were applied to the EEG data. Furthermore, EEG data were referenced to linked mastoids and downsampled to a sampling rate of 250 Hz. Afterwards, an independent component analysis was performed to remove eye blinks and other artifactual components, which then were removed semi manually using IClabel (Pion-Tonachini et al., 2019) and visual inspection. After removal, epochs with a time frame of 900 ms for the relevant segments (strong emotional positive, strong emotional negative, weak emotional positive, weak emotional negative) were cut with a pre-stimulus baseline of −100 ms before feedback presentation using ERPlab v8.30 (Lopez-Calderon and Luck, 2014). Epochs exceeding 75 μV were removed in an artifact rejection step. We calculated the peak-to-peak FRN by subtracting the positivity in the P2 time window from the negativity in the N2 time window at channel FCz (Ferdinand, 2019; Ferdinand and Hilz, 2020; Gheza et al., 2018; Paul et al., 2020). In line with the literature, we used slightly different time windows for younger and older adults to catch the peaks adequately (Luck and Gaspelin, 2017). Therefore, we used the negativity in a time window of 230–330 ms and the positivity in a time window of 170–230 ms for younger adults and the negativity between 240 and 340 ms and the positivity between 180 and 240 ms post feedback presentation for older adults. For both participant groups, the P3b was defined as the mean amplitude between 350 and 550 ms after feedback (Martín, 2012; Polich, 2007). In order to investigate potential age-related frontal shifts in the distribution of P3b activity, the component was analyzed at three midline electrode positions: Fz, Cz, and Pz.

### 2.5 Data analysis

To assess learning performance, we performed mixed ANOVAs with Age (young, old) as between-subject factor and Feedback Condition (strong emotional, weak emotional) and Learning Block (1,2,3,4) as within-subject factors on accuracy and reaction times as dependent variables. For all behavioral analyses, trials with reaction times < 100 ms were excluded. The analysis of reaction times further included only correct trials. Significant effects involving the factor Learning Blocks were followed up with pairwise comparisons between adjacent quarters (LB1 vs. LB2, LB2 vs. LB3, and LB3 vs. LB4) in order to limit the number of comparisons and maintain interpretability.

ERP analyses were carried out using a mixed-repeated measures ANOVA with Age (young, old) as between-subject factor, and Feedback Condition (strong emotional, weak emotional) and Valence (positive, negative) as within-subject factors. To be able to examine topographical differences in the P3b, the factor Channel (Fz, Cz, Pz) was additionally included in this analysis. To reduce the number of comparisons, it was entered into the ANOVA as a repeated contrast (Fz vs. Cz, Cz vs. Pz).

Even though we did not have an explicit hypothesis about ERP change over time, we included an additional factor Learning Half (1, 2) for the FRN *post-hoc* analysis, because a main effect of valence was not revealed in the original analysis, contradicting empirical evidence (Eppinger et al., 2008; Holroyd and Coles, 2002; Wurm et al., 2020). While the behavioral data were split into quarters, there were not enough trials to divide the ERP data in the same manner; therefore, the ERP data were split into halves. For younger adults, the mean number of included trials for strong emotional positive feedback was 130.73 (*SD* = 17.68) in the first learning half and 145.46 (*SD* = 11.88) in the second learning half. For strong emotional negative feedback, the corresponding means were 25.50 (*SD* = 12.09) and 12.96 (*SD* = 12.50). In the weak emotional condition, mean included trials for positive feedback were 116.46 (*SD* = 17.66) in the first learning half and 139.57 (*SD* = 16.72) in the second learning half, whereas mean included trials for negative feedback were 36.57 (*SD* = 17.76) and 18.62 (*SD* = 13.05), respectively. For older adults, the mean number of included trials for strong emotional positive feedback was 114.10 (*SD* = 16.66) in the first learning half and 135 (*SD* = 17.85) in the second learning half. For strong emotional negative feedback, mean included trials were 35.35 (*SD* = 14.79) and 21 (*SD* = 16.32). In the weak emotional condition, mean included trials for positive feedback were 92.04 (*SD* = 29.34) and 101.26 (*SD* = 27.38) for the first and second learning half, respectively, and mean included trials for negative feedback were 59.17 (*SD* = 18.88) and 49.48 (*SD* = 27.02).

For all statistical analyses, results were reported following a hierarchical approach. Specifically, when higher-order interactions involving a given factor were statistically significant, lower-order effects (i.e., main effects or lower-order interactions including the same factor) were only reported if they were not contradicted by these higher-order interactions. In cases where higher-order effects contradicted lower-order effects, only the more informative higher-order effects, followed by targeted follow-up analyses to clarify the nature of these interactions, were described. Accordingly, only those effects that remained interpretable in the absence of conflicting higher-order interactions were described. This reporting strategy was applied consistently across all analyses to ensure a clear and coherent interpretation of the results.

If necessary, Greenhouse-Geisser corrections were applied to adjust for sphericity violations, and epsilon-corrected *p*-values with uncorrected degrees of freedom are reported. For ANOVAs, we report partial eta squared as an effect size. Bonferroni corrections were used for *post-hoc* tests, and Cohen's *d* is reported for pairwise comparisons. The significance level was set to α = 0.05 for all analyses, which were conducted in R Studio Version 4.3.2 (R Core Team, R Foundation for Statistical Computing, Vienna, Austria).

## 3 Results

### 3.1 Behavioral data

#### 3.1.1 Accuracies

The analysis of the relative frequencies of correct responses revealed a main effect of Learning Block [*F*_(3, 141)_ = 169.36, *p* < 0.001, $\eta_{p}^{2}$ = 0.78], indicating increasing accuracy over time, and a main effect of Feedback Condition [*F*_(1, 47)_ = 75.32, *p* < 0.001, $\eta_{p}^{2}$ = 0.62], revealing better learning after strong than weak emotional feedback. In addition, there was a main effect of Age [*F*_(1, 47)_ = 19.88, *p* < 0.001, $\eta_{p}^{2}$ = 0.30], that was qualified by interactions with Feedback Condition [*F*_(1, 47)_ = 21.32, *p* < 0.001, $\eta_{p}^{2}$ = 0.31], and with Learning Block and Feedback Condition [*F*_(3, 141)_ = 5.14, *p* = 0.002, $\eta_{p}^{2}$ = *0*.10].

To resolve the three-way interaction, pairwise comparisons between adjacent learning blocks were conducted separately for each combination of Age and Feedback Condition. In younger adults, accuracy significantly increased from LB1 to LB3 after strong as well as weak emotional feedback [strong emotional: LB1 vs. LB2: *t*_(25)_ = −8.94, *p* < 0.001, *d* = −1.75; LB2 vs. LB3 *t*_(25)_ = −2.97, *p* = 0.006, *d* = −0.58; LB3 vs. LB4: *p* = 0.922; weak emotional: LB1 vs. LB2 *t*_(25)_ = −10.20, *p* < 0.001, *d* = −2.00; LB2 vs. LB3 *t*_(25)_ = −3.82, *p* < 0.001, *d* = −0.75; LB3 vs. LB4: *p* =0.234]. In older adults, the same pattern was found [strong emotional: LB1 vs. LB2: *t*_(22)_ = −10.02, *p* < 0.001, *d* = −2.09; LB2 vs. LB3: *t*_(22)_ = −2.30, *p* = 0.032, *d* = −0.48; LB3 vs. LB4: *p* = 0.408; weak emotional: LB1 vs. LB2: *t*_(22)_ = −2.20, *p* = 0.039, *d* = −0.46; LB2 vs. LB3: *t*_(22)_ = −2.28, *p* = 0.033, *d* = −0.48; LB3 vs. LB4: *p* = 0.502].

**Figure 2:**
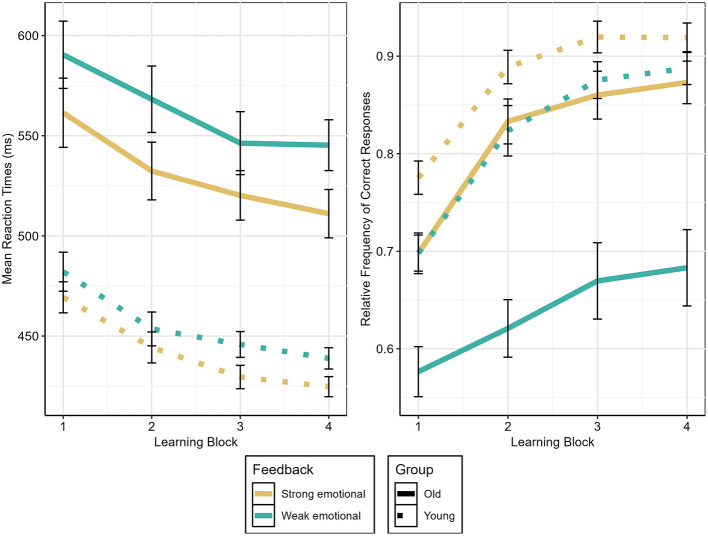
Mean reaction times and relative frequency of correct responses for both groups and both feedback conditions across four learning blocks. Whiskers denote standard error of the mean.

To explore age-related differences underlying the present three-way interaction, we examined accuracy across learning blocks (please see Figure 2). After strong emotional feedback, younger adults showed significantly higher accuracies than older adults in LB1 [*t*_(44)_ = −2.88, *p* = 0.006, *d* = −0.83]. In contrast, age differences did not reach statistical significance in LB2, LB3 or LB4 (all *p*-values >0.050). In the weak emotional feedback condition, younger adults consistently outperformed older adults across all learning blocks [LB1: *t*_(41.1)_ = −3.84, *p* < 0.001, *d* = −1.11; LB2: *t*_(45.3)_ = −5.17, *p* < 0.001, *d* = −1.48; LB3: *t*_(31.8)_ = −4.74, *p* < 0.001, *d* = −1.38; LB4: *t*_(30)_ = −4.81, *p* < 0.001, *d* = −1.40].

#### 3.1.2 Reaction times

The ANOVA on reaction times revealed a main effect of Age, with younger adults having faster reaction times than older adults [*F*_(1, 47)_ = 46.68, *p* < 0.001, $\eta_{p}^{2}$ = 0.49], and a main effect of Learning Block [*F*_(3, 141)_ = 42.07, *p* < 0.001, $\eta_{p}^{2}$ = 0.47], with decreasing reaction times from LB1 to LB4 [LB1 vs. LB2: *t*_(97)_ = 7.79, *p* < 0.001, *d* = 0.78; LB2 vs. LB3: *t*_(97)_ = 5.11, *p* < 0.001, *d* = 0.52; LB3 vs. LB4: *t*_(97)_ = 2.23, *p* = 0.028, *d* = 0.23]. Additionally, a main effect of Feedback Condition [*F*_(1, 47)_ = 64.95, *p* < 0.001, $\eta_{p}^{2}$ = 0.58] and an interaction between Age and Feedback Condition [*F*_(1, 47)_ = 10.88, *p* = 0.002, $\eta_{p}^{2}$ = 0.19] were revealed. Resolving the interaction resulted in both age groups having significantly faster reaction times after strong than weak emotional feedback [older adults: *t*_(91)_ = −8.58, *p* < 0.001, *d* = 0.89; younger adults: *t*(103) = −6.63, *p* < 0.001, *d* = 0.65], with a larger effect size for older than younger adults (see Figure 2).

### 3.2 Electrophysiological data

#### 3.2.1 Peak-to-peak FRN

**Figure 3:**
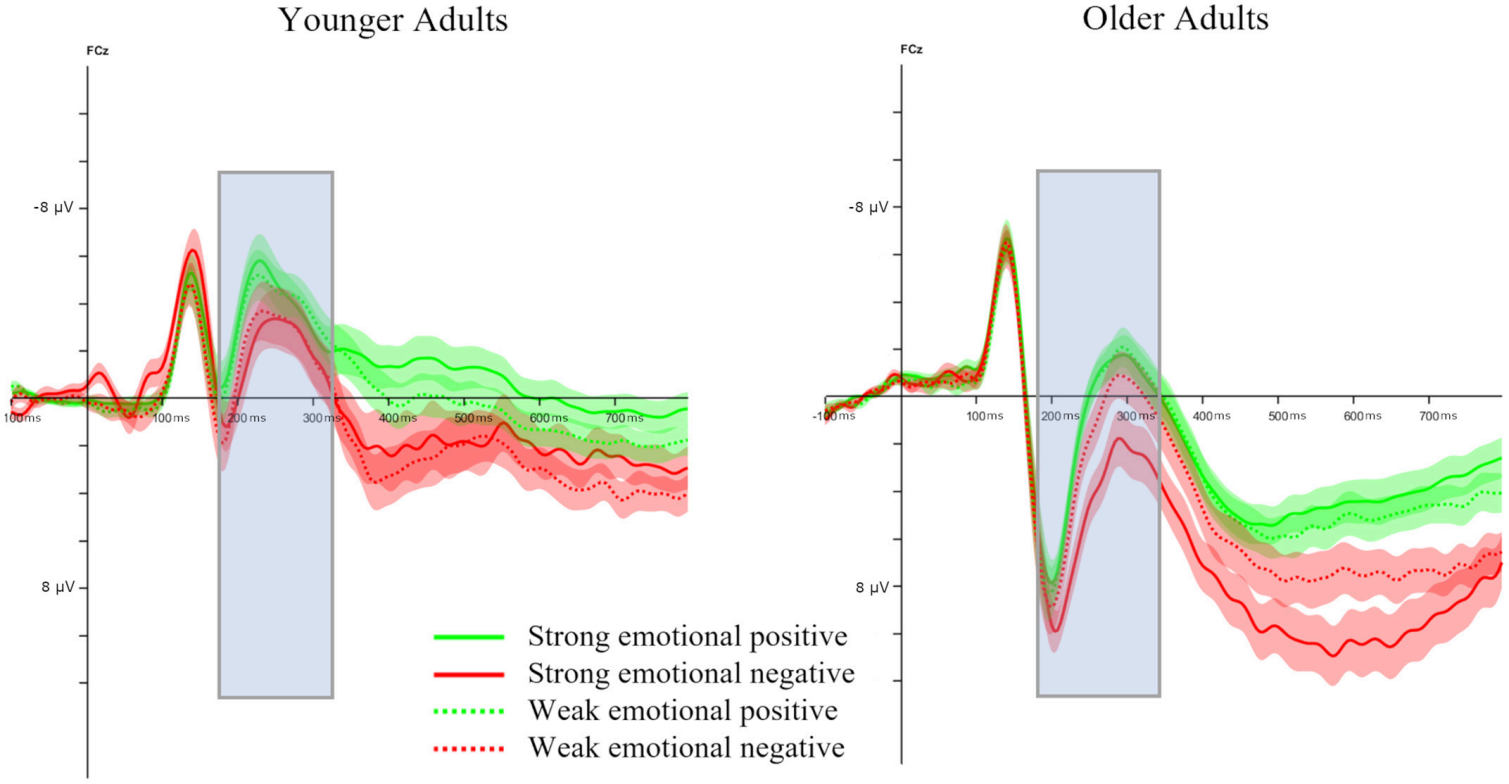
Feedback-locked ERP waveforms at channel FCz. Peak-to-peak FRN time range is marked and shaded error bands, representing the standard error of mean, are shown.

**Figure 4:**
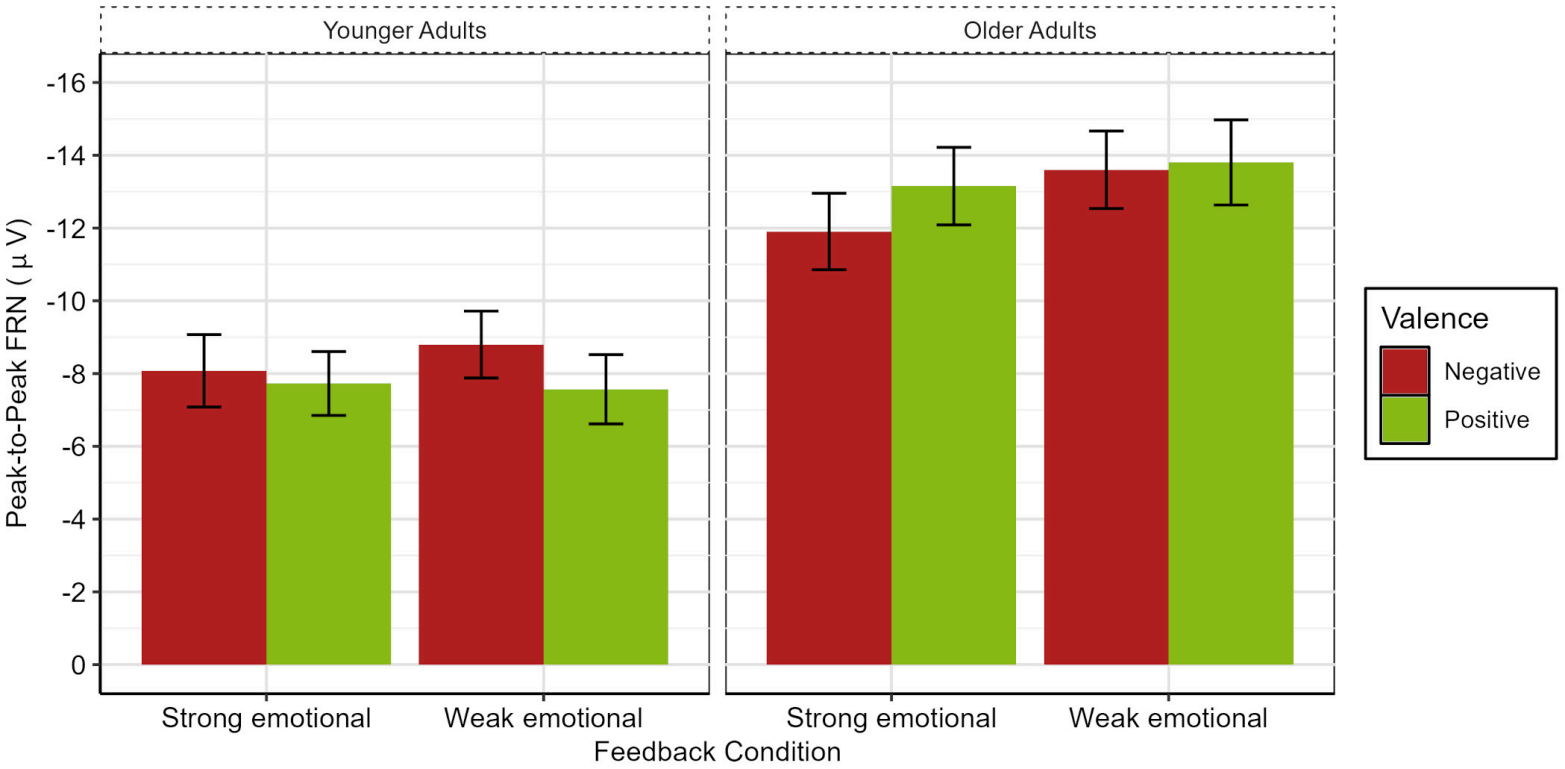
Bar plot illustrating the size of the peak-to-peak FRN at channel FCz. Error bars denote standard error of the mean.

The peak-to-peak FRN ANOVA model yielded an effect of Age, with older adults exhibiting a larger peak-to-peak FRN than younger adults [*F*_(1, 47)_ = 16.15, *p* < 0.001, $\eta_{p}^{2}$ = 0.25, see Figures 3, 4]. No other effects reached significance. Importantly, neither a significant main effect for Feedback Condition (*p* = 0.058) nor significant interactions with this factor were found (all *p*-values >0.09).

To better understand the absence of the typical FRN valence effect, we conducted *post-hoc* analyses by splitting the experiment into halves and including this factor in the mixed ANOVA. Doing so revealed again the main effect of Age [*F*_(1, 47)_ = 12.77, *p* < 0.001, $\eta_{p}^{2}$ = 0.21], an interaction between Age and Valence [*F*_(1, 47)_ = 7.80, *p* = 0.008, $\eta_{p}^{2}$ = 0.13], and Age, Valence and Learning Half [*F*_(1, 47)_ = 21.18, *p* < 0.001, $\eta_{p}^{2}$ = 0.31]. *Post-hoc* comparisons revealed age-related differences in FRN amplitudes that varied as a function of Valence and Learning Half. During the first learning half, older adults showed significantly larger FRN amplitudes than younger adults following both negative feedback [*t*_(87)_ = −4.98, *p* < 0.001, *d* = −1.01], and positive feedback [*t*_(83.9)_ = −4.72, *p* < 0.001, *d* = −0.96]. In the second learning half, no significant age difference was observed for negative feedback (*p* = 0.26). In contrast, for positive feedback, older adults continued to exhibit significantly larger FRN amplitudes than younger adults [*t*_(94.6)_ = −6.05, *p* < 0.001, *d* = −1.22].

**Figure 5:**
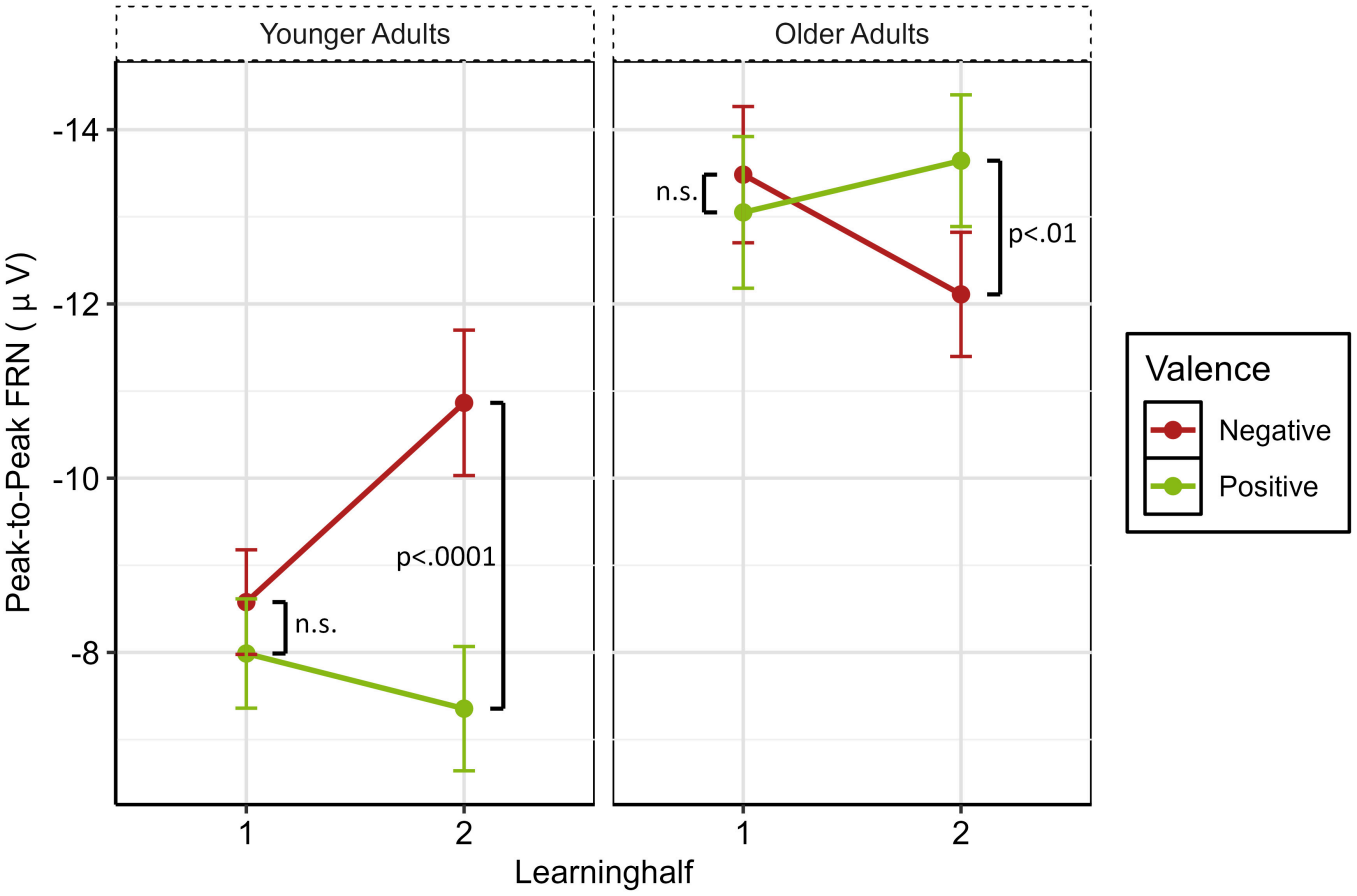
Bar plot illustrating the interaction between Age, Valence, and Learning Half. Error bars represent the standard error of the mean; n.s., not significant.

Further, we examined changes in peak-to-peak FRN amplitudes from Learning Half 1 to Learning Half 2 separately for each Age Group and Valence. In older adults, FRN amplitudes were significantly larger in Learning Half 1 than in Learning Half 2 following negative feedback [*t*_(45)_ = −3.29, *p* = 0.002, *d* = −0.27]. No significant change across learning halves was observed for positive feedback (*p* = 0.208). In Learning Half 1, there was no significant difference between positive and negative feedback for older adults (*p* = 0.395), while this age group showed a larger FRN after positive than negative feedback in Learning Half 2 [*t*_(45)_ = 2.91, *p* = 0.006, *d* = 0.31; see Figure 5]. In younger adults, FRN amplitudes differed significantly across learning halves following negative feedback, with larger amplitudes in Learning Half 2 than in Learning Half 1 [*t*_(51)_ = 2.65, *p* = 0.011, *d* = 0.44]. For positive feedback, the difference did not reach significance (*p* = 0.078). In addition, there was no significant difference between negative and positive feedback in Learning Half 1 for younger adults (*p* = 0.415), but a larger FRN after negative as compared to positive feedback in Learning Half 2 [*t*_(51)_ = −4.27, *p* < 0.001, *d* = −0.63; see Figure 5].

#### 3.2.2 P3b

**Figure 6:**
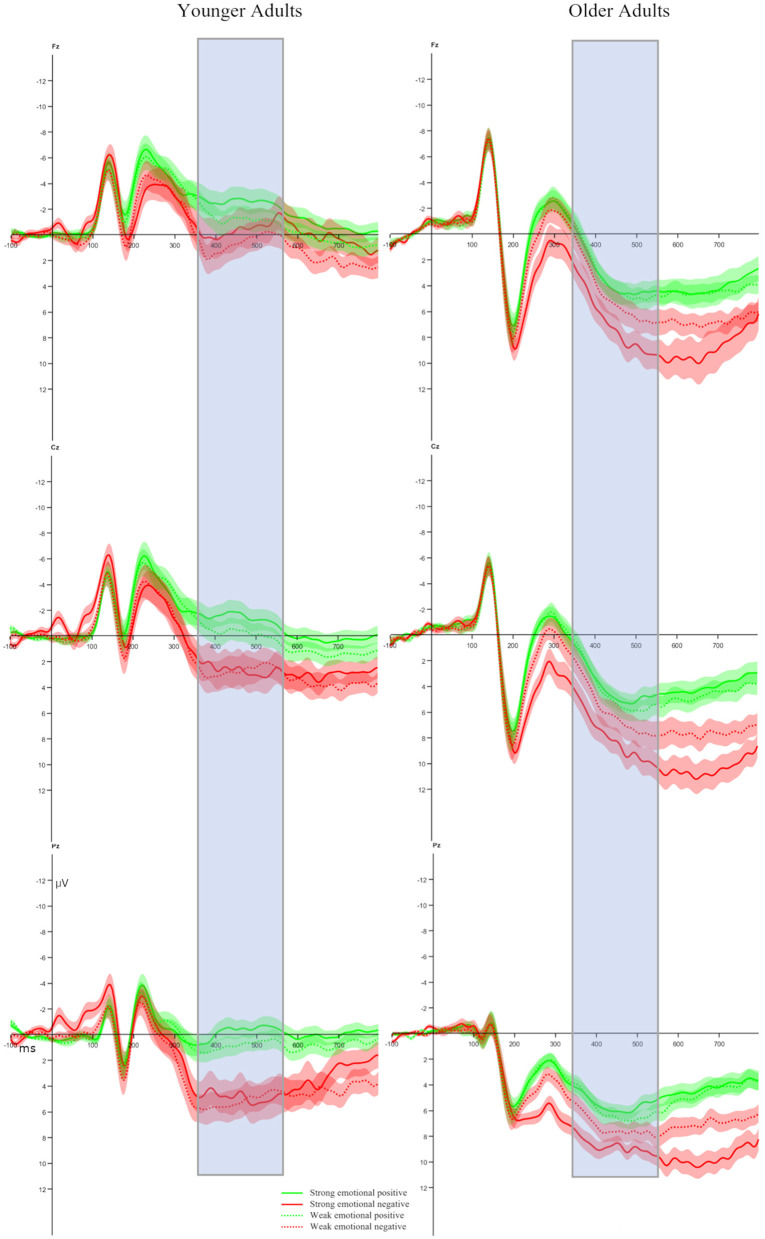
Feedback-locked ERP waveforms at three midline electrodes (Fz, Cz, Pz). Mean P3b time range is marked and shaded error bands represent the standard error of the mean.

**Figure 7:**
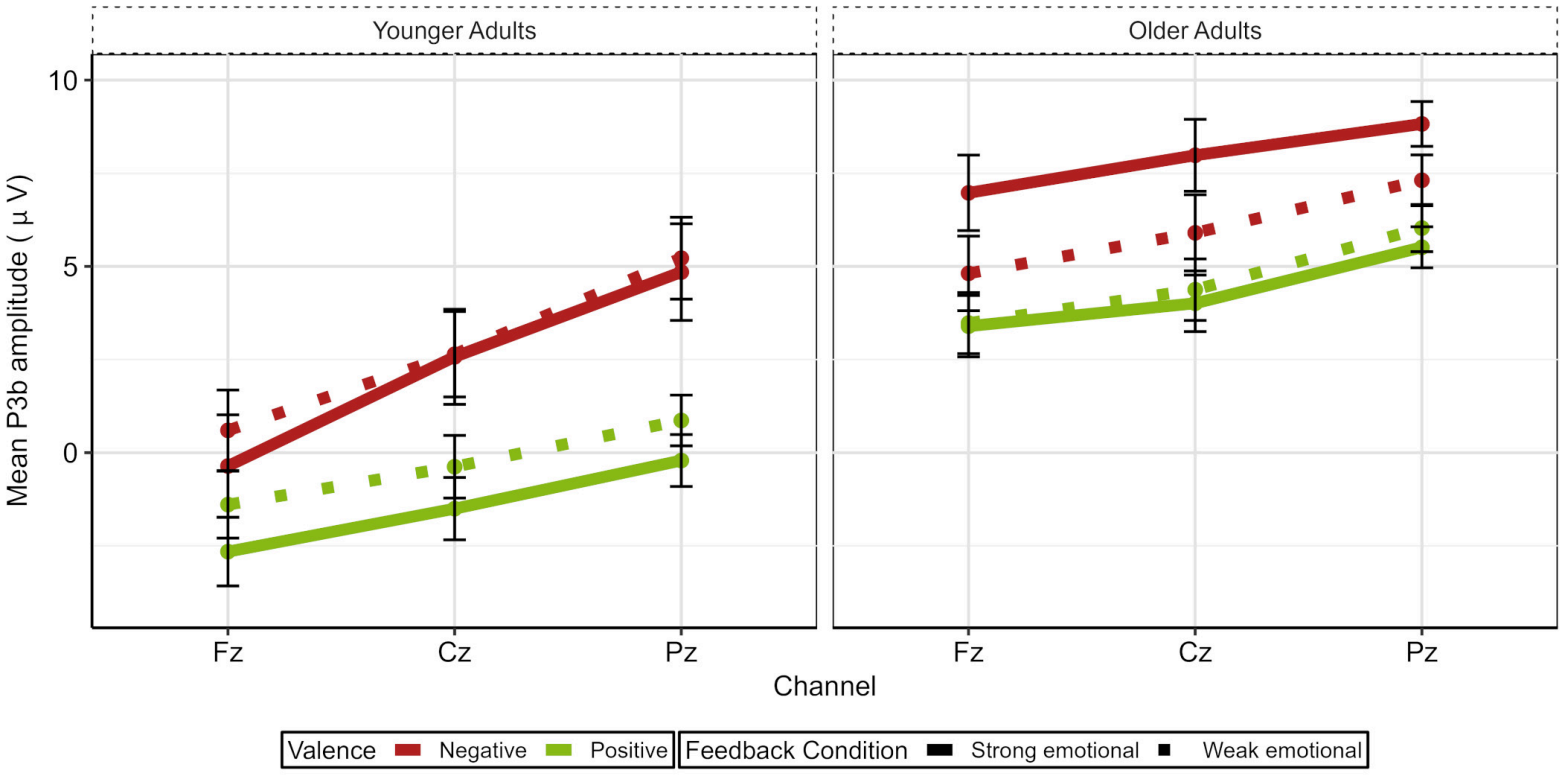
Line plot illustrating the P3b topography for both age groups. Error bars represent the standard error of the mean.

The P3b ANOVA model (please see Figures 6 and 7) yielded a significant main effect of Age [*F*_(1, 47)_ = 18.98, *p* < 0.001, $\eta_{p}^{2}$ = 0.29], with older adults showing larger mean P3b amplitudes than younger adults. In addition, main effects for Valence [*F*_(1, 47)_ = 54.16, *p* < 0.001, $\eta_{p}^{2}$ = 0.54] and Channel [*F*_(2, 94)_ = 25.79, *p* < 0.001, $\eta_{p}^{2}$ = 0.35] were observed. Further, two-way interactions between Age and Feedback Condition [*F*_(1, 47)_ = 7.68, *p* = 0.008, $\eta_{p}^{2}$ = 0.14], Valence and Feedback Condition [*F*_(1, 47)_ = 9.79, *p* = 0.003, $\eta_{p}^{2}$ = 0.17], and Valence and Channel [*F*_(2, 94)_ = 11.94, *p* < 0.001, $\eta_{p}^{2}$ = 0.20] were found. Last, interactions between Age, Valence, and Channel [*F*_(2, 94)_ = 14.67, *p* < 0.001, $\eta_{p}^{2}$ = 0.24] and Age, Feedback Condition, and Channel [*F*_(2, 94)_ = 3.64, *p* = 0.030, $\eta_{p}^{2}$ = 0.07] were revealed.

**Figure 8:**
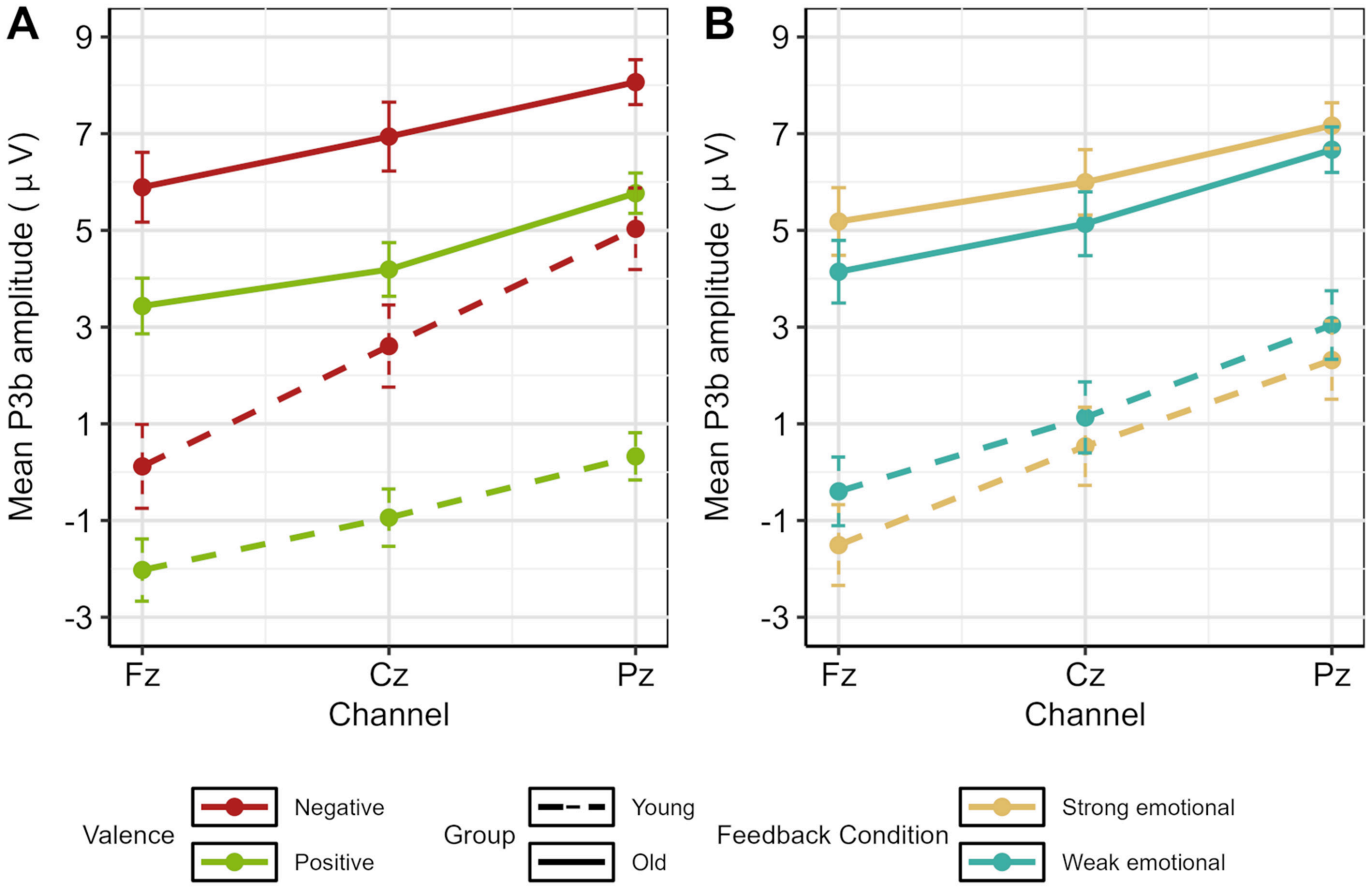
Bar plot illustrating **(A)** the interaction between Age, Valence and Channel and **(B)** Age, Feedback Condition and Channel. Error bars represent the standard error of the mean.

To investigate age-related differences between groups, we next analyzed the two three-way interactions including the factor Age. For the interaction between Age, Valence, and Channel (please see Figure 8A), significant age-related differences were found at all three electrodes for negative and positive feedback, resulting in older adults having larger P3b amplitudes than younger adults [Fz: negative: *t*_(94.60)_ = 5.11, *p* < 0.001, *d* = 1.03, positive: *t*_(95.80)_ = 6.33, *p* < 0.001, *d* = 1.27; Cz: negative: *t*_(94.80)_ = 3.91, *p* < 0.001, *d* = 0.78, positive: *t*_(96)_ = 6.32, *p* < 0.001, *d* = 1.27; Pz: negative: *t*_(78.40)_ = 3.16, *p* = 0.002, *d* = 0.63, positive: *t*_(95.10)_ = 8.48, *p* < 0.001, *d* = 1.70]. For the interaction between Age, Feedback Condition, and Channel, we found significant differences between younger and older adults in both feedback conditions at all three electrodes with older adults again showing larger P3b amplitudes. [strong emotional: Fz: *t*_(94.80)_ = 6.15, *p* < 0.001, *d* = 1.24; Cz: *t*_(94.80)_ = 5.18, *p* < 0.001, *d* = 1.04; Pz: *t*_(81)_ = 5.17, *p* < 0.001, *d* = 1.03, weak emotional: Fz: *t*_(95.90)_ = 4.73, *p* < 0.001, *d* = 0.95; Cz: *t*_(95.80)_ = 4.06, *p* < 0.001, *d* = 0.82; Pz: *t*_(86.70)_ = 4.26, *p* < 0.001, *d* =0.85, please see Figure 8B].

Next, guided by our hypotheses regarding age-specific effects, we conducted ANOVAs within age groups to examine the effects of valence and feedback condition. The analysis of younger adults resulted in significant main effects for Valence [*F*_(1, 25)_ = 29.64, *p* < 0.001, $\eta_{p}^{2}$ = 0.54] and Channel [*F*_(2, 50)_ = 17.29, *p* < 0.001, $\eta_{p}^{2}$ = 0.41], and an interaction between Valence and Channel [*F*_(2, 50)_ = 18.85, *p* < 0.001, $\eta_{p}^{2}$ = 0.43]. For negative as well as positive feedback, the mean P3b showed a parietal focus as it was significantly larger at Pz vs. Cz [negative: *t*_(51)_ = −4.73, *p* < 0.001, *d* = −0.66; positive: *t*_(51)_ = −3.35, *p* = 0.002, *d* = −0.47] and at Cz vs. Fz [negative: *t*_(51)_ = 7.04, *p* < 0.001, *d* = 0.98; positive: *t*_(51)_ = 4.18, *p* < 0.001, *d* = 0.58]. In addition, P3b amplitudes were larger for negative relative to positive feedback at all electrode sites [Fz: *t*_(51)_=3.96, *p* < 0.001, *d* = 0.55; Cz: *t*_(51)_ = 6.61, *p* < 0.001, *d* = 0.92; Pz *t*_(51)_ = 8.37, *p* < 0.001, *d* = 1.16] with effect sizes increasing from frontal to parietal electrode sites (see Figure 8A).

**Figure 9:**
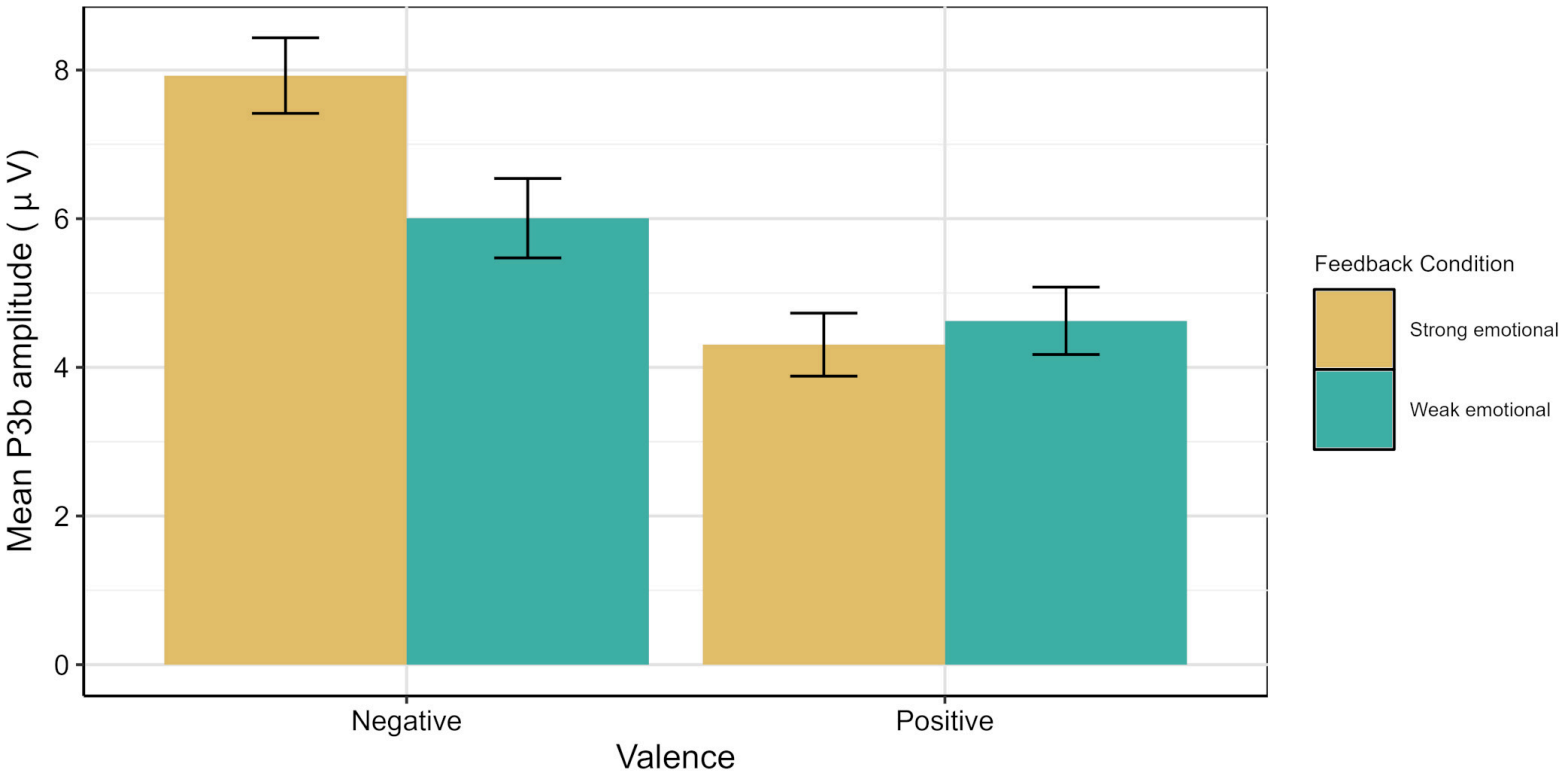
Mean P3b amplitude illustrating the interaction between Feedback Condition and Valence for older adults. Whiskers denote standard error of the mean.

The analysis of older adults revealed a significant main effect for Channel [*F*_(2, 44)_ = 9.14, *p* < 0.001, $\eta_{p}^{2}$ = 0.30], indicating a parietal distribution of the P3b for all conditions [Fz vs. Cz: *t*_(91)_ = 5.26, *p* < 0.001, *d* = 0.55; Cz vs. Pz: *t*_(91)_ = −4.75, *p* < 0.001, *d* = 0.49]. Moreover, a significant main effect for Valence [*F*_(1, 22)_ = 27.86, *p* < 0.001, $\eta_{p}^{2}$ = 0.56] and a significant interaction between Valence and Feedback Condition [*F*_(1, 22)_ = 12.25, *p* = 0.002, $\eta_{p}^{2}$ = 0.36] were revealed (please see Figure 9). After negative feedback, the mean P3b was larger after strong than weak emotional feedback [*t*_(68)_ = 5.13, *p* < 0.001, *d* = 0.62], whereas after positive feedback, no such difference was found (*p* = 0.202). Additionally, in both feedback conditions, the mean P3b amplitude was larger after negative than positive feedback [strong emotional:*t*_(68)_ = 10.2, *p* < 0.001, *d* =1.23; weak emotional: *t*_(68)_ = 4.19, *p* < 0.001, *d* = 0.50].

## 4 Discussion

In the present study, we investigated how different intensities of socio-emotional feedback influence learning in younger and older adults. We assumed that strong emotional feedback would support especially older adults to increase their learning performance. Furthermore, we examined whether strong emotional feedback would lead to an enhanced detection of unexpected outcomes, as reflected by the peak-to-peak FRN, and enhanced working memory updating, as indicated by the mean amplitude of the P3b. For this, participants performed a probabilistic learning task requiring them to learn stimulus–response associations, during which they received feedback through facial expressions conveying either weak or strong emotional feedback.

### 4.1 Learning performance

The present findings provide evidence that learning from feedback is affected by both age and intensity of socio-emotional feedback. While younger participants outperformed older adults in terms of faster reaction times, accuracy was influenced by the type of feedback, which was presented. Learning performance improved over time in both age groups with reaction times decreasing steadily across the task, indicating general learning through feedback. While both groups' accuracy also improved, younger adults exhibited a steeper learning curve and reached peak performance earlier, whereas older adults showed slower and more gradual improvements, particularly during the earlier learning phases. The type of feedback played a significant role in shaping learning performance. Across both age groups, strong emotional feedback led to higher accuracies and faster responses compared to weak emotional feedback. However, this benefit was particularly pronounced in older adults, as indicated by larger effect sizes for both accuracy and reaction times. The interaction between Age and Feedback Condition further supports the view that older adults profit disproportionately more from emotionally salient feedback (Ferdinand and Hilz, 2020). While both younger and older adults improved after strong emotional feedback, the gains were larger in the older group, suggesting that emotionally charged feedback may act as a motivational enhancer that reduces age-related learning deficits (Ferdinand and Hilz, 2020; Mather, 2012). Notably, following strong emotional feedback, the performance of older adults reached the level of younger adults under the same condition. This finding is particularly noteworthy in light of well-documented age-related performance declines (Eppinger et al., 2008; Ferdinand, 2019; Lighthall et al., 2018; Mell et al., 2005; Pietschmann et al., 2011; Wild-Wall et al., 2009). Visual inspection of the accuracy data further indicates that older adults in the strong emotional feedback condition performed comparably to younger adults in the weak emotional feedback condition. This pattern may reflect a motivationally driven benefit of emotional feedback by enhanced emotional engagement (Gorlick et al., 2013), enabling older adults to maintain cognitive control despite reduced learning rates.

At the same time, our findings add to prior evidence that younger adults can also benefit from socio-emotional feedback, for example in an item-category association task (Hurlemann et al., 2010) and probabilistic learning with varying task demands (Braunwarth and Ferdinand, 2025). However, in Ferdinand and Hilz (2020), younger adults did not show an accuracy benefit from socio-emotional compared to neutral feedback; their accuracy increased over the course of learning, but without significant differences between the feedback conditions. This observation suggests that the benefits of socio-emotional feedback in younger adults may have been masked by ceiling effects in that study. Altogether, these findings highlight the role of emotional intensity in feedback learning and demonstrate that emotionally relevant feedback can facilitate performance across the lifespan. In older adults in particular, preserved sensitivity to socio-emotional stimuli appears to enhance learning, underscoring the potential value of socio-emotional stimuli in cognitive interventions.

### 4.2 Detection of unexpected events

In the present study, we expected the detection of unexpected events to be reduced in older adults compared to younger adults, but at the same time having significant differences in the intensity of emotional feedback. Interestingly, older adults in our study exhibited larger peak-to-peak FRN amplitudes than younger adults. This finding deviates from previous studies reporting reduced performance monitoring and diminished FRN responses in older age, due to the age-related decline of dopaminergic signals (Bellebaum et al., 2012; Gajewski et al., 2018; Gajewski and Falkenstein, 2014; Hämmerer et al., 2011; Nieuwenhuis et al., 2005). However, our results are consistent with earlier work (Ferdinand and Hilz, 2020), in which older adults also showed enhanced detection of unexpected events following socio-emotional feedback (emotional vs. neutral faces), suggesting that socio-emotional feedback may preserve or even amplify the processing in older age. This could be grounded in motivational theories of aging, which imply that stimuli like faces, that can potentially convey socio-emotional information, might be valuable for shaping the motivation in performing a task successfully (Carstensen, 1991; Carstensen et al., 2006). However, this claim requires additional empirical support.

Unexpectedly, the effect of emotional intensity on the FRN was marginally non-significant. This is in contrast to a previous study, where socio-emotional feedback (emotional faces) led to a stronger processing of unexpected events in younger adults as compared to non-emotional feedback (scrambled faces; Braunwarth and Ferdinand, 2025). One possible explanation is that our study used faces with weak emotional expressions as the comparison condition, whereas the previous study employed scrambled faces, which differed more explicitly in emotional content. We used weak emotional expressions to create a comparison condition that matched the strong expressions on perceptual features while differing in intensity only. Nonetheless, despite the pronounced behavioral effects, the rapid detection mechanism reflected in the peak-to-peak FRN may not have been sufficiently sensitive to capture these more subtle variations in emotionality.

We also did not find a significant effect of feedback valence, which is typically observed during feedback-induced learning when participants form outcome predictions (Holroyd and Coles, 2002; Martín, 2012; Paul et al., 2025). A reason for this could be that the valence effect changes with increasing learning. Thus, we conducted *post-hoc* analyses taking learning progress into account. However, the trial numbers for negative feedback within the *post-hoc* analysis were low, therefore the results should be interpreted with caution. Nevertheless, these analyses showed that a valence differentiation arose with learning in younger adults, who exhibited a stronger FRN to negative feedback in the second half of the experiment. This is in line with the idea that negative feedback should become more unexpected the more participants have learned (Holroyd and Coles, 2002; Miltner et al., 1997). Older adults likewise showed no valence effect in the first half of learning. In the second half, however, they exhibited a more negative FRN to positive than to negative feedback, reflecting the opposite pattern of younger adults. This possibly indicates an age-related shift in the importance of positive feedback. Prioritizing positive information aligns with the well-documented positivity effect in aging (Mather and Carstensen, 2005; Reed et al., 2014) and has also been proposed in earlier studies on feedback-induced learning (Bellebaum et al., 2012; Eppinger et al., 2008). However, evidence for this effect remains inconsistent (Ferdinand, 2019; Ferdinand and Kray, 2013; Fernandes et al., 2018).

Taken together, our findings suggest that, compared with younger adults, older adults show enhanced detection of unexpected events when feedback is socio-emotional in nature, potentially reflecting age-related motivational shifts in feedback processing. While younger adults' monitoring system is especially sensitive for negative feedback once learning has been established, older adults show the opposite pattern, consistent with a positivity effect.

### 4.3 Working memory updating

The present findings revealed clear age-related differences in working memory updating, as indexed by the P3b. Interestingly, older adults showed larger P3b amplitudes than younger adults, suggesting enhanced updating mechanisms, which, however, have usually been found to be decreased with age in similar tasks (Ferdinand, 2019; Ferdinand and Kray, 2013; Schmitt et al., 2014a; West and Huet, 2020). One possible explanation for this could be that in our study, all of the feedback stimuli consisted of facial expressions, which due to their socio-emotional character may have a specific impact on the activation of working memory updating especially in older age. To our knowledge, the only other study examining feedback processing in the P3b in older and younger adults using emotional faces is the study by Ferdinand and Hilz (2020). In this study, however, age-differences in P3b amplitudes were not explicitly tested. Descriptively, however, P3b amplitudes for older adults did not seem to be enhanced in the emotional condition as compared to younger adults. Thus, it remains an open question for future research which precise feedback stimulus characteristics can lead to larger P3b amplitudes in older adults.

In both younger and older adults, the P3b showed the expected parietal maximum, consistent with its role in updating working memory representations in response to task-relevant feedback (Gaál and Czigler, 2015; Martín, 2012; Polich, 2007). In older adults, however, we assumed that the distribution over the scalp would be broader, with an enhanced frontal activation, likely reflecting the compensatory recruitment of additional resources to maintain effective updating when processing feedback (Cabeza et al., 2002; Daffner et al., 2011; Ferdinand, 2019; Ferdinand and Hilz, 2020; Friedman et al., 1997; Lubitz et al., 2017; Pietschmann et al., 2011; Reuter-Lorenz et al., 2000; Schmitt et al., 2014a,b; Tregellas et al., 2006; Wild-Wall et al., 2009). One possible explanation is that the feedback stimuli were clearly interpretable, given that both feedback conditions used similar facial expressions differing only in emotional intensity and that the task itself was comparatively easy. Nevertheless, a possible replication of these findings is an open topic for future research.

Across both age groups, working memory updating was enhanced for negative relative to positive feedback. This finding indicates that negative feedback elicited a stronger updating of task-relevant representations, consistent with the requirement to adapt behavior after errors in probabilistic learning tasks (Ferdinand, 2019; Ferdinand and Hilz, 2020; Frank et al., 2005; Kamp et al., 2024; but see Braunwarth and Ferdinand, 2025). Among younger adults, we observed a clear valence effect that was not modulated by emotional intensity. One possible explanation is that efficient updating processes in this age group operate independently of the emotional intensity of feedback. It also replicates the results of Ferdinand and Hilz (2020), who found that socio-emotional feedback *per se* did not enhance working memory updating in younger adults, but negative feedback did.

In the present study, we found no main effects or interactions involving emotional intensity in younger adults. However, this is in contrast to Braunwarth and Ferdinand (2025), who did report effects of emotionality for younger adults. One possible account of this finding is that in the present study both feedback conditions were socio-emotional in nature, as they both contained faces with weak and strong emotional expressions, whereas in Braunwarth and Ferdinand (2025) one condition consisted of scrambled faces and thus most likely lacking most of their socio-emotional aspects. Therefore, the latter study provided a more explicit contrast, potentially increasing the relevance of socio-emotional feedback in working memory updating.

In contrast, and most notably, older adults exhibited a marked effect of emotional intensity in the age-specific analyses: working memory updating following negative feedback was significantly larger for strong than for weak emotional feedback. This finding replicates the results of Ferdinand and Hilz (2020), and supports the notion that socio-emotional feedback benefits older adults' working memory updating. However, an alternative explanation could be that older adults may have greater difficulty differentiating feedback valence when emotional intensity is weak, possibly due to subtle changes in emotion perception with age (Ruffman et al., 2008). Nevertheless, this interpretation is challenged by the fact that learning performance after weak emotional feedback was comparable to earlier studies such as the neutral and socio-emotional feedback conditions in Ferdinand and Hilz (2020; both below and around 0.7 relative frequency of correct responses in last learning quarter) and that performance after strong emotional feedback was exceptionally high. This high performance may also be related to the choice of happiness and disgust as feedback emotions, since recognition of these expressions is usually well-maintained in older adults (Orgeta and Phillips, 2007; Ruffman et al., 2008).

Therefore, we assume that strong emotional feedback can substantially enhance working memory updating in older adults by activating the dopaminergic system, compensating for age-related decline (Baeckman et al., 2000; Bäckman et al., 2006).

### 4.4 Limitations and outlook

One possible limitation of the present study could be that the age of participants who rated the emotional intensity and valence of feedback in the preliminary study was significantly younger (mean age = 24.39, range= 18–56 years) than those included in the older age group of the present study. This may have resulted in older adults recognizing the feedback stimuli less well than younger adults. Consequently, the stimuli may not have been equally valid for different age groups, which could lead to an overestimation of socio-emotional feedback processing and how effectively older adults can learn from it. However, one could argue that such discrepancies are meaningful in themselves, since older adults are likely to have different thresholds for interpreting emotional signals. Furthermore, learning performance in the weak emotional condition was consistent with earlier studies. In the strong emotional condition, however, we observed a steep increase, possibly due to the use of happy and disgusted facial expressions. Nevertheless, future research should aim to validate feedback stimuli for different age groups. Potential ceiling effects in younger adults may have limited observable improvements in the benefit of socio-emotional feedback during learning as well. Moreover, we cannot determine whether the socio-emotional benefit observed in this study is driven by the emotional expression of the faces, the social nature of the faces themselves, or the inherent communicative meaning of emotional expressions. Therefore, future research could benefit from developing feedback stimuli that can distinguish between these different options. This would not only increase methodological precision, but also provide deeper insights into how socio-emotional feedback is used by older adults during learning.

### 4.5 Conclusion

In this study, we found that both groups learning performance benefited from strong emotional feedback, while the effect was particularly pronounced for older adults. Interestingly, older adults showed a generally stronger detection of unexpected events, as reflected in the FRN, which we assume might be due to the socio-emotional content of the feedback stimuli we used. Our findings further suggest that strong socio-emotional feedback strengthens working memory updating in older adults and, under optimal conditions, may enable older adults to reach performance levels comparable to those of younger adults. This has important implications for developing targeted cognitive training programs, refining feedback strategies, and designing evidence-based interventions that strengthen learning in older adults, thereby promoting autonomy and sustained engagement in everyday life.

## Data Availability

The datasets presented in this study can be found in online repositories. The names of the repository/repositories and accession number(s) can be found below: https://doi.org/10.17605/OSF.IO/QM37X.
